# A prospective study of the role of sleep related disordered breathing as a risk factor for motor vehicle crashes and the development of systemic complications in non-commercial drivers

**DOI:** 10.1186/1749-7922-9-2

**Published:** 2014-01-07

**Authors:** Eric D Irwin, Patricia Reicks, Alan Beal, Matthew Byrnes, Craig Matticks, Greg Beilman

**Affiliations:** 1 3300 Oakdale Ave, Robbinsdale, MN 55422, USA; 2 1116 Gillespie, Garden City, KS, USA

**Keywords:** Sleep related disordered breathing, Motor vehicle crashes

## Abstract

**Introduction:**

Sleep related disordered breathing (SRDB), is an established risk factor for motor vehicle crashes (MVCs) involving commercial drivers. The role of SRDB in motor vehicle crashes involving non-commercial drivers is not well established.

**Methods:**

Drivers involved in MVCs who were admitted to an American College of Surgeons accredited Level I trauma center for treatment of their injuries, and who could give informed consent and provide verbal responses to screening questionnaires were eligible for enrolment in this study. Two questionnaires previously validated for screening patients at risk for sleep disturbances (The Epworth Sleepiness Scale (ESS) and The Berlin Questionnaire (BQ)) were administered. Questionnaire results associated with an 85% sensitivity for predicting obstructive sleep apnea were considered positive. In this study we tested the hypothesis that patients at risk for SRDB, as measured by validated questionnaires, are at an increased risk being involved in MVCs.

**Results:**

Between March and October 2010, 71 consecutive patients were offered enrolment in this study with 56 agreeing to participate in this study. Six were previously diagnosed with SRDB with only one being compliant and effectively treated at the time of their MVC. Forty-two patients (75%) had responses to the questionnaires that indicated that the patients were at high risk for SRDB. Six patients suffered systemic complications, including pleural effusions, pneumonia and arrhythmias, during their hospitalization with five (83%) having abnormal questionnaire responses indicating that the patient was at high risk for SRDB.

**Conclusions:**

The high incidence of positive responses to the sleep questionnaires is consistent with the hypothesis that SRDB is potentially a significant risk factor for MVCs. Furthermore the observation that systemic complications were seen more commonly in those with SRDB, while not unexpected, is a novel observation. Further studies are needed to validate these findings in a larger cohort of patients as well as determining if these patients are truly at a greater risk for systemic complications. If replicated these observations would suggest that effective therapy for disordered sleep could play a significant role in an injury prevention process.

## Introduction

The significance of Sleep Related Disordered Breathing (SRDB) as a contributing factor in motor vehicle crashes (MVCs) has been reported for commercial drivers [1-3]. Additionally, an increased risk of MVCs has been reported in patients undergoing formal sleep studies as part of their workup for obstructive sleep apnea [4]. Little, however, has been written about the role of SRDB as a risk factor for MVCs in non-commercial drivers who do not carry a formal diagnosis of SRDB.

SRDB constitutes a spectrum of conditions characterized by repeated episodes of hypopnea (under breathing), which is reported in up to 25% of all adult males and 9% of all adult females [5], to more extreme respiratory effort-related arousals such as advanced obstructive sleep apnea (OSA), which is reported to affect 5% of the general population [6]. These disorders may result in hypoventilation and reduced gas exchange as well as systemic organ dysfunction and daytime sleepiness, fatigue or inattention. These latter symptoms have been shown to predispose to occupational injuries [7] and increased motor vehicle crashes (MVCs) in operators of commercial motor vehicles [1-3] and in an older series of a general population of patients involved in MVCs [8]. Additionally Turkington and colleagues found that patients with OSA, who were tested in driving simulators, demonstrated behaviors that were significantly more likely to result in MVCs [9].

SRDB has also been shown to have systemic effects including; activation of the sympathetic nervous system [10], activation of the coagulation system [11], activation of the renin-angiotensin-aldosterone system [12,13] and altered endothelial function [14]. These observations help explain why SRDB is a risk factor for medical conditions including hypertension [15], acute coronary syndromes [16], virulent atherosclerosis [15], cardiac arrhythmias [15], and systemic inflammatory conditions [14]. These latter effects may well predispose patients with sleep-related disorders who sustain injuries to suffer increased rates of complications.

This study was undertaken to further investigate the possible relationship between patients at risk for SRDB and motor vehicle crashes in non-commercial drivers. Additionally, given the systemic effects of SRDB a secondary objective was to evaluate for a possible association between SRDB and the development of complications in a consecutive series of non-commercial operators of motor vehicles who required admission for the treatment of injuries sustained in a MVC.

## Methods

Approval was obtained from the local Institutional Review Board and all research was performed in compliance with the principles in the Declaration of Helsinki. Patients admitted to an American College of Surgeons Level I trauma center, who were operators of motor vehicles involved in crashes, were offered entry into the study. The study limited entry to individuals who were able to provide informed consent and answer the sleep questionnaires. Patients agreeing to participate in the study were evaluated using two previously validated questionnaires, the Berlin questionnaire (BQ) and the Epworth Sleepiness Scale (ESS), to identify patients at risk for SRDB [17-23].

The BQ was developed by the Conference on Sleep in Primary Care. Questions were selected from the literature to elicit factors or behaviors that consistently predicted the presence of SRDB [17-20]. The instrument focused on a limited set of known risk factors for sleep apnea. The results of the BQ are presented as high or low probability of SRDB, The responses to these questions have been shown to have utility in non–primary care settings [17]. Other information was obtained about hypertension, age, weight, height, and gender and neck circumference. Obesity was quantified by calculating body mass index from self-reported weight and height.

The ESS was developed by Johns et. al. at the Sleep Disorders Unit of the Epworth Hospital in Melbourne Australia. This questionnaire rates the chances that a patient will ‘doze off’ or ‘fall asleep’ when in eight different situations commonly encountered in daily life. This was initially presented in 1991 when it was reported that Total ESS scores distinguish normal subjects from patients in various diagnostic groups including obstructive sleep apnea syndrome, narcolepsy and idiopathic hypersomnia [21]. Further validation was provided when ESS scores were found to correlate significantly with sleep latency measures, a measure of how quickly one falls asleep in quiet situations during the day. This correlation was demonstrated during multiple sleep latency tests and during overnight polysomnography. In overnight polysomnography patients are monitored using electroencephalography (EEG), electrooculography (EOG), and surface electromyography (EMG) and recordings made to identify patterns of physiologic variables that may be used to diagnose abnormalities of sleep and/or wakefulness and other physiologic disorders that have an impact on or are related to sleep and/or wakefulness. [21]. Unlike the BQ, which provides a result of high or low risk for SRDB, the ESS provides a numeric output ranging from 0 to 24. In studies across a range of patient groups responses to ESS were considered ‘Normal Sleep’ (ESS = 1–6), ‘Average Sleepiness’ (ESS = 7–8) and ‘Abnormal Sleep’ (ESS = 9–24) [21-23]. The questionnaires are presented in Additional file 1.

After completing the questionnaires, neck measurements were performed as described by Davies et. al. [24]. Demographic data were subsequently obtained from the trauma registry and electronic medical record.

Overall data is divided into normal and abnormal groups based on their specific questionnaire responses and presented by study questionnaire and each of these groups pooled. For the purposes of pooling, the normal group was defined as those patients with a BQ ranking of low risk for SRDB and an ESS of < 9. The abnormal group was defined as those patients with a BQ ranking of high risk for SRDB and/or an ESS score of ≥ 9.

Statistical analysis for differences between groups was performed using Analyze-it statistical software and was performed using a *t*-test for groups for parametric analysis and a Mann–Whitney Rank Order test for nonparametric analysis. Data are presented as Mean ± Standard Deviation. Differences between groups for a given variable were considered significant if the p value was < 0.05.

## Results

Over the five-month period of time during which this study was conducted 1,830 patients were admitted to the ACS level I verified trauma center. Of these 71 operators of vehicles who could provide informed consent and responses to the questionnaires were identified and offered enrolment in this study, of these 56 patients agreed to participate. Patient demographics for the group as a whole as well as by questionnaire results are presented in Table 1. Patients with an abnormal response on BQ or ESS are referred to as ‘Positive’ with the remaining patients categorized as ‘Normal’. Data are presented for these groups in Table 2. Patients with positive questionnaire responses were statistically significantly older and heavier than those with normal responses. In contrast neck circumference was not different between the groups in terms of absolute measurement or measurement normalized by body height. In the six patients with a positive admission measurement for blood alcohol, the ESS was ≥9 in four patients and BQ responses were in the high risk of SRDB in three patients.

**Table 1:** Patient Demographics

	**All**	**Normal BQ**	**Abnormal BQ**	**Normal ESS**	**Abnormal ESS**
Gender (M: F)	41:15	17:8	24:7	10:4	31:11
Age (Years)	45.6 ± 13.2	42.5 ± 12.8	48.2 ± 13.2	43.3 ± 14.5	46.4 ± 12.8
Height (cm)	175.6 ± 10.4	173.3 ± 10.3	177 ± 10.1	171.3 ± 10.6	177 ± 9.9
Weight (kg)	94.8 ± 24.8	80.33 ± 14.4	106.5 ± 25.3	76.8 ± 13.8	100.8 ± 24.8
BMI	30.6 ± 7.34	26.5 ± 4.13	33.9 ± 7.71	26.0 ± 4.76	32.5 ± 8.41
Neck circumference*	42.37 ± 5.02	40.3 ± 4.67	44 ± 4.76	40.8 ± 4.76	42.8 ± 5.06
Normalized neck circumference**	0.24 ± 0.024	0.23 ± 0.022	0.25 ± 0.02	0.238 ± 0.022	0.242 ± 0.024

*Neck circumference in centimeters.

**Normalized neck circumference = neck circumference in centimeters divided by height in centimeters.

**Table 2:** Demographic Data for patients with normal and abnormal questionnaire responses

	**Normal**	**Abnormal**
Gender (M:F)	10:4	31:11
Age (Years)	42.17 ± 13.81	47.3 ± 12.78^1^
Height (cm)	172.6 ± 10.3	177.3 ± 10^1^
Weight (kg)	78.22 ± 12.85	102.7 ± 25.24^1^
BMI	26.1 ± 4.06	32.7 ± 7.51^1^
Neck circumference*	40.89 ± 4.35	43.1 ± 5.23^1^
Normalized neck circumference**	0.238 ± 0.02	0.242 ± 0.025^1^
Serum Na	139.1 ± 1.96	139.8 ± 2.59^1^
Serum Cr	0.98 ± 0.24	0.99 ± 0.24
ETOH positive	0	6
		Range 106-338

*, ** indicates that the differences between the groups was stastically significant.

The group of patients who screened positive for SRDB, based on questionnaire results, had a lower mean Injury Severity Score (ISS). Despite this they had longer Intensive Care Unit (ICU) and hospital stays and an increase in number of ventilator days and complications Table 3. Six patients experienced complications. These complications included arrhythmias, a transfusion reaction, respiratory failure requiring reintubation and bleeding in one patient each and pleural effusions in two patients. These patients had an average ISS = 22.3 ± 8.8, which was significantly greater than that from the group not experiencing complications. Five of the six patients had BQ survey results putting them at high risk of SRDB; however their ESS scores were statistically equivalent to the group that did not experience any complications. While the small number of patients precludes meaningful statistical analysis two thirds of the complications involved the cardiovascular system or inflammatory processes which are consistent with known effects of sleep disordered breathing.

**Table 3:** Hospitalization and complication data

	**Normal**	**Abnormal**
Hospital LOS	3.06 ± 2.44	5.16 ± 5.26^1^
ICU LOS	0.22 ± 0.647	1.5 ± 2.44^1^
Vent days	0	0.45 ± 1.27^1^
Deaths	0	0
ISS	10.17 ± 8.28	9.81 ± 8.04^1^
Any complications	0	6^1^

^1^Differences significant for *t*-test p < 0.05.

Most crashes occurred during weekdays rather than weekends. The distribution throughout the time of day showed the greatest number of crashes occurring between 12 noon and 6 P.M. for both weekday and weekend crashes and for those with normal or abnormal questionnaire results.

With respect to medications that were being taken by the patients on entry to this study. One patient in each group was on a medication for preventing seizures. A seizure was implicated as the cause of the MVC in the patient with a positive survey response. This patient is one of the patients who developed systemic complications from injuries sustained in the MVC. Additionally; three patients in the abnormal group and one patient in the normal sleep group were being treated for diabetes at the time of their MVC. Hypoglycemia was not implicated as a contributing factor in any of the MVCs. Five patients in the abnormal sleep group were taking opioids at the time of their admission (Oxycodone N = 2 and Hydrocodone, Oxycontin and Methadone in one patient each). There had been no recent additions to the medication regimens of these patients and none had experienced changes in their health resulting in an increased use of opioids immediately preceding their MVC.

## Discussion

We have demonstrated that a significant number of operators of motor vehicles involved in MVCs report findings on the Berlin Questionnaire and the Epworth Sleepiness Scale that are consistent with Sleep Related Disordered Breathing (SRDB). This observation is consistent with that reported in studies of commercial drivers [1,2,6,9] but is at variance with the findings in non-commercial drivers presented by Teran-Santos and associates, who did not find a relationship between ESS scores and the risk of MVCs [8].

Howard et al. found a significant relationship between ESS scores and the risk for a MVC in operators of commercial vehicles, with the odds ratio of being involved in a MVC increasing from 1.5 for an ESS of 8–10, (mild OSA), to 1.91 in patients with an ESS score of 18–24, (severe OSA) [1]. They also observed that people with more severe symptoms (ESS = 19–24) were more likely to have been involved in multiple MVCs (odds ratio = 2.67 (1.29-5.52)) [1]. Shiomi studied patients admitted to a sleep disorders clinic for daytime sleepiness and found that an ESS > 11 was associated with moderate to severe OSA and that the rate of MVCs over a five year period of time was 3.8% in patients who were simple snorers, to 5.8%, 9.9% and 11% respectively for patients with mild, moderate and severe OSA [6]. Similar observations were made by others reporting results in commercial drivers [2,3,6,9]. Given the structure of our study we cannot quantify risk, however, our findings do support the relationship between SRDB and MVCs demonstrated by the above authors in commercial drivers and expands this finding to non-commercial drivers.

Our results are similar to those reported by Barbe et al. in a study of patients undergoing formal sleep studies, where a mean ESS score of 12 was reported for those involved in MVCs compared to a mean ESS score of 3 for controls, not involved in MVCs. Furthermore, those with abnormal ESS scores were not only more likely to have been in a single MVC but were more likely to be involved in multiple MVCs with an odds ratio of 5.2. These differences persisted even after stratification for mileage driven per year, alcohol consumption and age [4]. An ESS of ≥ 12 was seen in 15 of our patients.

In our study patients with positive questionnaire results had a significantly greater weight and BMI as compared to patients with normal questionnaire results (P < 0.05). They also had a greater actual and normalized neck circumference, although these differences did not reach statistical significance. Increased BMI and neck circumference are both factors that have been associated with OSA in study populations [24], and in studies of SRDB as a risk factor for MVCs in commercial drivers [2,4].

In a recent study of commercial drivers in the United Kingdom, Horne and colleagues reported that the risk of MVCs, attributed to SRDB, peaks between 0200–0600 and 1400–1600 (Horne et. al. [25]. In contrast to these findings, in our series only 7 crashes occurred between the hours of 02:00 and 06:00, four with abnormal ESS and four with High-risk BQ results. Additionally, nine crashes occurred between the hours of 14:00 and 16:00 in our patient cohort. Thus, the temporal distribution of crashes throughout the weekend/weekday or time of day was more evenly distributed in our patients than has been described in studies of commercial drivers. While the structure of our study does not allow formal hypothesis-testing, these observations are consistent with the hypothesis that SRDB is associated with an increased risk for sleepiness and inattention [7] and may have effects that extend throughout the day, not only during times in which fatigue would be expected to be most pronounced.

In our study only 6 patients (10.7%) had positive screens for alcohol. This is consistent with the findings of Longo et al. who found that 12.6% of drivers of vehicles involved in crashes were intoxicated at the time of their crash [26]. It is, however, below the rate of 17% for all trauma-related admissions at our institution during the same time period [unpublished data]. Again these observations are consistent with the notion that SRDB is a contributing factor for MVCs in non-commercial operators of motor vehicles.

Many common medications have been implicated as contributing to SRDB. These include beta-adrenergic antagonists, oral hypoglycemic agents, angiotensin converting enzyme inhibitors, antidepressants and benzodiazepines [4]. Barbe et. al. found that these were being taken by seven of 60 patients (12%) with OSA who were involved in MVCs, as compared to one of 60 control patients [4]. Our findings are at variance with their observation as these agents were being taken by an equivalent percentage of patients in each group. In nine patients (21%) with abnormal sleep questionnaires, and four of 14 patients (28%) with normal sleep questionnaire responses. Four patients had the diagnosis of diabetes on admission with hypoglycemia not being implicated in any cases. Five patients had a history of documented seizure disorder and were under treatment at the time of their MVC. A seizure was implicated as causing the MVC in only one of our cases. This patient was in the abnormal group and their hospital course was remarkable for developing pleural effusions.

The BQ has not been used as extensively as the ESS in assessing the risk of SRDB in operators of motor vehicles. In our series 25 patients gave responses to the BQ questionnaire that indicated a higher risk of SRDB, 22 of these patients also had ESS scores indicating a high risk of SRDB. A larger sample size would be required before a more detailed analysis of the individual elements of the questionnaires can be performed.

A total of six patients suffered complications during their post-injury course. Five of these patients had responses on their BQ indicating a high risk of SRDB while the ESS results were statistically equivalent to those who did not suffer systemic complications of their injuries. The small sample size of this study precludes formal statistical analysis of the questionnaires individual elements and their association with systemic complications. Admittedly, the ISS results for these patients were greater than that of the patient cohort overall, which would be expected to have contributed to these complications. Given the reports of SRDB being associated with activation of the sympathetic nervous system [10], activation of the coagulation system [11,27] with alterations in systemic inflammatory processes [14], and endothelial function [14,27], the possible role of SRDB as a contributing factor in complications following trauma warrants further investigation.

The main limitations of this study are its small sample size and only enrolling those who were less severely injured and could provide their own responses to the SRDB questionnaires. The small sample size precludes us from formally testing hypotheses. This study does, however, provide data about the relationship between SRDB and noncommercial drivers and is the basis for a larger multicenter study that has been initiated. If these observations are repeated in this larger study it would provide a straightforward means of identifying patients at an increased risk of developing systemic complications during the time that they are hospitalized for the treatment of their injuries. Secondly by excluding patients with more severe injuries, and who were unable to provide their own responses prevents us from making any conclusions about this more severely injured group of patients.

## Conclusions

In summary, we have found that non-commercial operators of motor vehicles involved in crashes have a higher prevalence of positive responses to questionnaires developed to identify patients with increased daytime sleepiness and a variety of forms of SRDB. Additionally, although the sample size was small, precluding analysis of the effects of all confounding factors, we have found that patients who suffered complications following MVCs were more likely to have responses on their Berlin Questionnaires indicating a high risk for SRDB. Further studies are required to confirm the relationship between SRDB and MVCs and between SRDB and the development of systemic complications in patients who sustain trauma.

## Abbreviations

(BQ): Berlin questionnaire; (EGG): Electroencephalography; (EOG): Electrooculography; (EMG): Electromyography; (ESS): Epworth sleepiness scale; (ICU): Intensive care unit; (ISS): Injury severity score; (MVC): Motor vehicle crash; (OSA): Obstructive sleep apnea; (SRDB): Sleep related disordered breathing.

## Competing interests

All authors declare that they have no competing interests.

## Authors’ contributions

EI participated in study design, data acquisition and analysis and manuscript preparation. PR participated in data acquisition and analysis and manuscript preparation. AB participated in data analysis and interpretation and manuscript preparation. MB participated in study design, statistical analysis, data interpretation and manuscript preparation. CM participated in data interpretation and manuscript preparation. GB participated in study design, data interpretation and manuscript preparation. All authors read and approved the final manuscript.

## Supplementary Material

Additional file 1 Sleep questionnaires. Click here for file
