# Supplementary material for: A prospective study of the role of sleep related disordered breathing as a risk factor for motor vehicle crashes and the development of systemic complications in non-commercial drivers
Source: World J Emerg Surg. 2014 Jan 7;9:2. doi: 10.1186/1749-7922-9-2 (PMC3933287; doi:10.1186/1749-7922-9-2)
Supplement: Additional file 1 — Sleep questionnaires. [file 1749-7922-9-2-S1.doc]

**Table S1. Sleep Questionnaires**

| **Epworth Sleepiness Scale – For Patient** |  |
| --- | --- |
| **SITUATION (refers to your usual way of life in recent times. Even if you have not done some of these things recently, try to work out how the would have affected you).** | 0 = would never doze  1 = Slight chance of dozing  2 = Moderate chance of dozing  3 = High chance of dozing |
| Sitting and reading |  |
| Watching TV |  |
| Sitting, inactive, in a public place |  |
| As a passenger in a car for an hour |  |
| Lying down in the afternoon |  |
| Sitting and talking to someone |  |
| Sitting quietly after a lunch without alcohol |  |
| In a car, while stopped for a few minutes in traffic |  |
| **TOTAL** |  |

How likely are you to doze off or fall asleep in the following situations? (In contrast to just feeling tired)

Berlin Questionnaire

1. Do you Snore?
   - Yes
   - No
   - Do not know
2. If you Snore:
3. Your snoring is?
   - 1. Slightly louder than breathing
     2. As loud as talking
     3. Louder than talking
     4. Very loud, Can be heard in adjacent rooms.
4. How often do you snore?
   - 1. Nearly every day
     2. 3-4 times a week
     3. 1-2 times a week
     4. 1-2 times a month
     5. Never or nearly never
5. Has your snoring ever bothered other people?
   - 1. 1) Yes 2) No
6. Has anyone noticed that you quit breathing during your sleep?
   - 1. Nearly every day
     2. 3-4 times a week
     3. 1-2 times a week
     4. 1-2 times a month
     5. Never or nearly never
7. How often do you feel tired or fatigued after you sleep?
   - 1. Nearly every day
     2. 3-4 times a week
     3. 1-2 times a week
     4. 1-2 times a month
     5. Never or nearly never
8. During your wake time, do you feel tired, fatigued or not wake up to par?
   - 1. Nearly every day
     2. 3-4 times a week
     3. 1-2 times a week
     4. 1-2 times a month
     5. Never or nearly never
9. Have you ever nodded off or fallen asleep while driving a vehicle?
   - 1. Yes
     2. No
     3. If yes, how often does it occur
        1. Nearly every day
        2. 3-4 times a week
        3. 1-2 times a week
        4. 1-2 times a month
        5. Never or nearly never
